# Respiratory ventilation modes in selective ophthalmic arterial chemotherapy for pediatric retinoblastoma under general anesthesia

**DOI:** 10.3389/fmed.2026.1791936

**Published:** 2026-04-15

**Authors:** Yuxing Wang, Xiang Dong, Weiling Gao, Jiegang Dong, Xu Wang, Jie Chen, Hong Jiang

**Affiliations:** Department of Anesthesiology, Shanghai Ninth People’s Hospital, Shanghai Jiao Tong University School of Medicine, Shanghai, China

**Keywords:** intra-arterial chemotherapy, mechanical ventilation, ophthalmic artery, pediatric anesthesia, retinoblastoma

## Abstract

**Objective:**

To determine the optimal ventilation strategy for pediatric patients with retinoblastoma undergoing selective ophthalmic arterial chemotherapy (SOAC) by comparing the intraoperative effects of volume-controlled (VCV), pressure-controlled (PCV), and pressure-controlled volume-guaranteed (PCV-VG) ventilation.

**Methods:**

105 children (aged 1.5–5 years) undergoing SOAC were assigned to three groups: VCV (*n* = 33), PCV (*n* = 37), and PCV-VG (*n* = 35). Hemodynamics, airway pressures, end-expiratory carbon dioxide (EtCO_2_), arterial blood gases, and clinical complications were recorded and analyzed.

**Results:**

Postoperatively, the peak airway pressure in Group V was significantly higher than those in Group P and Group G. The EtCO_2_ of Group P was closer to normal values than that of the other two groups during surgery *p* < 0.05. Blood gas results at the three-time points showed that pH and HCO_3_^−^ values of Group P were closer to normal than those of the other two groups immediately after the operation and 5 minutes after the end of angiography, respectively *p* < 0.05. No significant differences of other indicators were found among the three groups.

**Conclusion:**

Altogether, compared with the VCV, both PCV and PCV-VG modes produced beneficial lower peak airway pressures during SOAC, thus conducing to prevent mechanical ventilation lung injury.

## 1 Introduction

Retinoblastoma is a common primary intraocular malignant tumor, accounting for approximately 2–4% of childhood malignancies (1). If left untreated, the mortality rate can be as high as 95% (2, 3). Compared with the orthodox treatment for retinoblastoma, local chemotherapy (intraocular injection and interventional chemotherapy) may provide a better prognosis for children with insensitive or advanced retinoblastoma (4). With the advent of fluoroscopy and the maturity of neurovascular access technology, selective ophthalmic arterial chemotherapy (SOAC) has become an important technique for the treatment of retinoblastoma with low toxicity and few side effects (5). It can also significantly reduce the rate of eyeball removal and the adverse effects on visual acuity (6).

During SOAC, the insertion of an interventional catheter can irritate the intracranial vascular system, leading to changes in cardiorespiratory compliance in children, with an incidence that is as high as 24% (7, 8). An article published in 2013 reported that during the catheterization of the ophthalmic artery, patients exhibited a carbon dioxide waveform resembling bronchospasm, along with a rapid decrease in tidal volume. Additionally, there were occurrences of hypotension, bradycardia, and reduced lung compliance, which may be related to the trigeminal cardiac reflex and the ophthalmic reflex (9). Due to the special physiological and anatomical characteristics of children, as well as various adverse complications such as reduced lung compliance caused intraoperatively by SOAC, it may be particularly important to choose the appropriate ventilation mode.

Volume-controlled ventilation (VCV), pressure-controlled ventilation (PCV) and pressure-controlled volume-guaranteed ventilation (PCV-VG) are three widely utilized ventilation modes (10, 11). Among these, VCV can effectively maintain a constant tidal volume (12), however, airway pressure is affected by changes in resistance and lung compliance, which may lead to barotrauma in pediatric patients. PCV can set the airway pressure and inspiratory time in advance so that the airway pressure quickly reaches the preset value during inspiration (13), however elevated airway pressure may hinder the ability to guarantee tidal volume, potentially leading to inadequate ventilation during surgical anesthesia (14). PCV-VG is a new type of general anesthetic ventilation, combining the advantages of volume- and pressure-controlled ventilation. The PCV-VG mode calculates lung compliance to deliver the target tidal volume at the lowest possible pressure, effectively minimizing the risk of barotrauma during mechanical ventilation, reducing the incidence of postoperative atelectasis, and exerting a minimal impact on respiratory mechanics (12, 15, 16). Recent studies have found that the PCV-VG mode can effectively reduce airway pressure during mechanical ventilation in newborns, reduce the occurrence of neonatal lung injury, and provide lung protection in infants under anesthesia. Therefore, we hypothesize that during SOAC surgery, when lung compliance varies, the PCV-VG mode can provide more stable ventilatory support with a lower risk of barotrauma, while minimally impacting oxygenation, hemodynamics, and blood gas analysis.

This study aims to compare the changes in respiratory parameters and vital signs in children undergoing SOAC surgery under three ventilation modes: VCV, PCV, and PCV-VG. The primary objective is to determine which ventilation mode provides better lung protection and exerts a lesser impact on respiratory physiology, guiding the selection of the optimal ventilation strategy. The secondary objective is to compare the effects of these three ventilation modes on hemodynamics, analyze potential complications, and evaluate their impact on postoperative recovery.

## 2 Methods

### 2.1 Study design

This study is a retrospective analysis, and the requirement for informed consent from guardians has been waived. The study was conducted in accordance with the principles of the Declaration of Helsinki and was approved by the Ethics Committee of the affiliation in December 2020 (approval number: SH9H-2020-C47-2). The study documented children who underwent ultra-SOAC at the hospital. The study received the clinical trial registration number (NCT04990271) in December 2020.

### 2.2 Setting and participants

The infants who received SOAC between July 2020 and August 2021 were, respectively, collected in this study. The inclusion criteria were as follows: (1) age 1.5–5 years old; (2) American Society of Anesthesiologists (ASA) physical status II or III; (3) receiving VCV, PCV, or PCV-VG during general anesthesia. And the exclusion criteria for subjects collected in this study were listed as follows: (1) chronic diseases of other systems; (2) acute upper respiratory tract infection, or poorly controlled asthma; (3) contrast agent allergy; (4) difficult airways; (5) incomplete data. The subjects were divided into VCV (Group V), PCV (Group P), and PCV-VG (Group G) based on the ventilation modes. It is important to note that this was a purely retrospective observational study without any active randomization or intervention by the investigators. The choice among the three ventilation modes (VCV, PCV, or PCV-VG) was based entirely on the clinical preference of the attending anesthesiologist managing each specific case.

All infants were induced by general anesthesia with conventional tracheal intubation and fasted for solids for 6 h and liquids for 2 h before surgery. Intramuscular injection of atropine 0.01 mg/kg 0.5 h before surgery. The patients were administered an intramuscular injection of 5 mg/kg or an intravenous injection of 0.5–1 mg midazolam and carried into the operating room after falling asleep. Routine preoxygenation was performed after entering the room, and a multifunctional monitor was connected to monitor noninvasive arterial blood pressure, heart rate, pulse oxygen saturation (SpO_2_), and electrocardiogram. Before endotracheal intubation, full mask oxygen was administered for 2 min, followed by intravenous injection of dexamethasone 0.1 mg/kg, midazolam 0.1 mg/kg, fentanyl 2 μg/kg, propofol 2.5 mg/kg, rocuronium 0.6 mg/kg, rapid induction endotracheal intubation was performed, and endotracheal intubation was completed under the guidance of video laryngoscope (model). All infants were ventilated using the same mechanical ventilator (Datex-Ohmeda-Avance CS2 anesthesia machine; GE Healthcare, Madison, WI, USA). After the completion of general anesthesia intubation, a 24G trocar was inserted into the left dorsalis pedis artery to monitor the intraoperative invasive arterial pressure and get the blood gas analysis.

Ventilation settings for all groups were as follows: fresh gas flow rate: 1.5 L/min, oxygen concentration: 0.4–0.6, inspiratory/expiratory ratio: 1:2, end-expiratory carbon dioxide (EtCO_2_) concentration maintained at 35–45 mmHg, respiratory rate 20–30 times/min. The three different ventilation modes were shown as follows: (1) The Group V was mechanically ventilated in volumetric control mode with the tidal volume set at 6–7 mL/kg. (2) The Group P underwent mechanical ventilation in the pressure-controlled mode. The tidal volume was first set at 7 mL/kg, and the peak inspiratory pressure (PIP) was set in the same pressure-controlled mode based on the resulting peak airway pressure. (3) The Group G adopted a preset tidal volume of 7 mL/kg, a respiratory rate of 16–20 times/min, and a volume assurance pressure limit of 18 cmH_2_O.

The adequate depth of general anesthesia during the procedure was maintained using propofol 4–6 mg/kg/h and inhaling sevoflurane concentrations of 1.5–2.5%, BIS (Philips Healthcare, Andover, MA) at 40–60, remifentanil at 0.05–0.2 μg/kg/min.

Different time points were set for data collection: T0 (immediately after intubation), T1 (immediately after surgery beginning), T2 (immediately after microcatheter placement), T3 (immediately after contrast agent injection), T4 (2 min after contrast agent injection), T5 (5 min after contrast agent injection), T6 (30 min after contrast agent injection), and T7 (immediately after the end of operation). Vital signs were collected and recorded at the above time points, and blood gas was collected at T1, T5, and T7 for blood gas analysis.

The heart rate (HR), non-invasive blood pressure (NIBP), invasive arterial blood pressure (ABP), oxygen saturation (SpO_2_), end-expiratory CO_2_ (EtCO_2_), peak airway pressure (PIP) were monitored continuously during surgery, and blood gas parameters at the three time points: immediately after induction (A1), immediately after SOAC (A2), and immediately after surgery (A3). The operative duration (from the start to the end of the surgery), ventilation duration (from the completion of intubation to extubation), and extubation time (from the cessation of anesthesia to extubation) were recorded.

Meanwhile, adverse events such as skin flushing, urticaria, significantly increased airway peak pressure (>20 cmH_2_O), perioperative hypoxemia (SpO_2_ < 90%), intraoperative bradycardia, and intraoperative invasive arterial pressure reduction (<20% of the baseline value) were all recorded during the operation. Adverse events such as the use of cardiovascular active drugs (e.g., epinephrine) and intraday Surgical Intensive Care Unit (SICU) admission were recorded.

### 2.3 Statistical analysis

All statistical analyses were conducted using SPSS version 25.0 software (IBM Corp., Armonk, NY, USA). Demographic data are expressed as chi-square test rates or percentages, and chi-square tests were used to detect differences between groups. The measurement data are presented as mean ± standard deviation. The Shapiro–Wilk method was used to test the normality of the variables, and the normal distribution was consistent with *p* > 0.05. When the normal distribution was satisfied, the difference between groups was tested by one-way analysis of variance (F test). When the measured data did not meet the normal distribution, the difference between groups was detected by non-parametric Kruskal-Willis test. As this was a retrospective study, the sample size was determined by the number of eligible patients available during the study period. To assess the adequacy of the available sample size, a post-hoc power analysis was performed based on the peak airway pressure at the critical surgical time point (T3). Using the observed effect size among the three groups (η^2^ = 0.19) and a significance level of *α* = 0.05, the total sample size of 105 patients yielded an estimated statistical power of approximately 0.99.

## 3 Results

### 3.1 Baseline characteristics

Based on the inclusion and exclusion criteria, 105 children were enrolled in this study, and divided into three groups: VCV (Group V, 33), PCV (Group P, 37), and PCV-VG (Group G, 35). Their baseline indicators including age, sex, mean body weight, intraocular pressure, vasoactive agent application, operative duration, ventilation duration and extubation time were collected and analyzed, no significant differences of the baseline indicators were found among the three groups *p* > 0.05 (Table 1).

**Table 1:** Baseline data characteristics.

Variables	Group V	Group P	Group G	*p-*value	Statistic (F/χ^2^)
Age (month)	30.03 ± 13.78	33.65 ± 13.09	29.03 ± 11.9	0.284	1.275
Sex (Male/Female)	16/17	24/13	21/14	0.368	2.001
Weight (Kg)	13.74 ± 4.26	14.09 ± 2.54	13.41 ± 2.83	0.677	0.391
Eye (L/R/Double)	15/15/3	20/16/1	14/21/0	0.213	5.826
Respiratory Rate (breaths/min)	18.94 ± 1.95	18.89 ± 2.16	19.09 ± 2.28	0.924	0.079
Treated intraocular pressure(mmHg)	12.34 ± 3.98	14.39 ± 7.56	13.39 ± 8.29	0.479	0.741
Untreated intraocular pressure(mmHg)	11.39 ± 2.89	13.06 ± 6.16	11.00 ± 2.91	0.113	2.230
Vasoactive agent (no/yes)	27/6	30/7	28/7	0.982	0.037
Operative duration (min)	64.1.3 ± 21.1	59.3 ± 16.2	66.8 ± 22.5	0.277	1.301
Ventilation duration (min)	85.3 ± 17.1	79.6 ± 20.8	81.2 ± 22.7	0.492	0.715
Extubation time(min)	14.7 ± 3.9	13.1 ± 4.2	15.2 ± 3.4	0.058	2.932

Values are mean ± SD or median [IQR]. VCV, volume-controlled ventilation; PCV-VG, pressure-controlled volume-guaranteed ventilation; PCV, pressure-controlled ventilation.

### 3.2 P_peak_ and ΔP_peak_ among the three groups at different time points

We further compared the peak airway pressure (P_peak_) and differences in peak airway pressure (ΔP_peak_) among the three groups at different time points. The results exhibited that the P_peak_ was significantly lower in Group P and Group G than in Group V at T2-T7 (all *p* < 0.05). Compared to T1, T3 had a significantly elevated P_peak_ in Group V and the lowest P_peak_ was in Group P. However, ΔP_peak_ in the three groups showed no differences at T3-T2, T4-T2, T5-T2.

Among them, eight patients in Group V showed increased peak airway pressure (PIP > 20 cmH_2_O), two at time point T2, five at time point T3, and one at time point T5. An increase in airway pressure occurred in two cases in the Group P, both at T3. Airway pressure increased in seven patients in the Group G: one at T2, two at T3, three at T4, and one at T5. The immediate airway pressure (T0) after intubation was less than 20 cmH_2_O in all groups (Table 2).

**Table 2:** Comparison of peak airway pressure and differences in peak airway pressure among the three groups at different time points.

Time points	Group V	Group P	Group G	*P-*value	Statistic (F/χ^2^)
T0 peak	13.42 ± 2.19^a^	12.65 ± 1.11^a^	12.80 ± 1.76^a^	0.160	1.864
T1 peak	16.97 ± 2.56^b^	14.70 ± 2.11^a^	14.60 ± 1.70^a^	0.000	13.284
T2 peak	18.00 ± 3.48^b^	14.73 ± 2.12^a^	15.34 ± 3.25^a^	0.000	11.593
T3 peak	19.72 ± 5.68^b^	14.95 ± 2.52^a^	16.37 ± 4.58^a^	0.000	10.558
T4 peak	19.58 ± 5.99^b^	15.33 ± 3.00^a^	17.26 ± 5.19^a^	0.002	6.579
T5 peak	19.48 ± 4.80^b^	15.43 ± 3.39^a^	16.40 ± 4.11^a^	0.000	9.089
T6 peak	19.55 ± 4.98^b^	15.68 ± 3.46^a^	17.11 ± 3.78^a^	0.001	7.904
T7 peak	18.97 ± 2.95^b^	15.78 ± 3.50^a^	16.60 ± 2.58^a^	0.000	9.262
∆(T3-T2)	1.72 ± 6.07^a^	0.22 ± 1.32^a^	1.03 ± 4.87^a^	0.644	0.442
∆(T4-T2)	1.58 ± 5.65^a^	0.54 ± 2.32^a^	1.91 ± 5.59^a^	0.439	0.830
∆(T5-T2)	1.48 ± 4.77^a^	0.70 ± 3.37^a^	1.06 ± 4.61^a^	0.747	0.293

P_peak_, peak airway pressure; ΔP_peak_, peak airway pressure difference. Different superscript letters indicate significant differences between groups, *p* < 0.05.

### 3.3 Hemodynamics indicators and EtCO_2_ among the three groups

Hemodynamic indicators including MAP, HR and EtCO_2_ were also investigated in the present study. The results showed that the EtCO_2_ in Groups V and G showed a gradual decline over time. The EtCO_2_ in Group P remained at approximately 40 mmHg, which was a statistically significant difference compared with the other two groups *p* < 0.05. No significant difference in other indicators was found among the three groups (Table 3).

**Table 3:** Comparison of hemodynamic indicators in the three groups at different time points.

Variables	Group V	Group P	Group G	*P-*value	Statistic (F/χ^2^)
T0					
MAP (mmHg)	82.39 ± 9.27^a^	83.73 ± 13.29^a^	83.29 ± 13.10^a^	0.897	0.108
HR (bpm)	131.79 ± 13.77^a^	128.62 ± 13.33^a^	133.89 ± 14.10^a^	0.265	1.345
EtCO_2_ (mmHg)	43.18 ± 5.03^a^	42.19 ± 4.90^a^	42.63 ± 5.79^a^	0.733	0.312
T1					
MAP (mmHg)	64.55 ± 6.82^a^	63.00 ± 7.79^a^	61.29 ± 7.14^a^	0.213	1.570
HR (bpm)	121.67 ± 21.56^a^	120.86 ± 14.29^a^	127.66 ± 12.49^a^	0.171	1.799
EtCO_2_ (mmHg)	40.15 ± 5.69^a^	39.36 ± 5.61^a^	39.22 ± 8.27^a^	0.824	0.194
T2					
MAP (mmHg)	65.27 ± 7.37^a^	63.69 ± 7.36^a^	63.11 ± 6.00^a^	0.442	0.823
HR (bpm)	121.12 ± 16.26^a^	119.00 ± 14.30^a^	124.06 ± 12.64^a^	0.333	1.110
EtCO_2_ (mmHg)	38.96 ± 6.30^a^	39.14 ± 5.76^a^	38.57 ± 4.94^a^	0.910	0.094
T3					
MAP (mmHg)	64.03 ± 7.69^a^	63.25 ± 8.27^a^	63.34 ± 7.01^a^	0.906	0.099
HR (bpm)	119.48 ± 14.25^a^	118.59 ± 13.27^a^	120.60 ± 12.24^a^	0.814	0.206
EtCO_2_ (mmHg)	37.96 ± 5.94^a^	38.68 ± 8.21^a^	36.97 ± 5.52^a^	0.557	0.589
T4					
MAP (mmHg)	62.43 ± 10.02^a^	60.75 ± 7.18^a^	61.62 ± 7.90^a^	0.717	0.334
HR (bpm)	118.61 ± 13.67^a^	117.62 ± 11.94^a^	122.37 ± 14.81^a^	0.298	1.224
EtCO_2_ (mmHg)	37.92 ± 6.35^a^	40.18 ± 9.49^a^	36.51 ± 5.48^a^	0.109	2.266
T5					
MAP (mmHg)	60.06 ± 12.76^a^	59.03 ± 9.19^a^	56.32 ± 15.04^a^	0.183	1.728
HR (bpm)	119.67 ± 14.65^a^	115.49 ± 21.13^a^	122.63 ± 11.48^a^	0.183	1.728
EtCO_2_ (mmHg)	37.70 ± 6.84^a^	40.02 ± 9.65^a^	36.18 ± 5.68^a^	0.106	2.297
T6					
MAP (mmHg)	60.48 ± 12.58^a^	61.00 ± 9.04^a^	59.06 ± 8.89^a^	0.716	0.335
HR (bpm)	115.82 ± 12.61^a^	116.41 ± 13.07^a^	119.60 ± 13.03^a^	0.426	0.861
EtCO_2_ (mmHg)	37.76 ± 6.55^a^	41.31 ± 8.23^b^	35.64 ± 6.02^a^	0.004	5.978
T7					
MAP (mmHg)	62.42 ± 13.67^a^	61.11 ± 8.15^a^	59.38 ± 6.74^a^	0.458	0.786
HR (bpm)	114.00 ± 12.18^a^	143.68 ± 182.93^a^	117.94 ± 13.54^a^	0.461	0.779
EtCO_2_ (mmHg)	37.71 ± 6.78^ab^	39.86 ± 7.38^b^	35.91 ± 5.43^a^	0.043	3.243

MAP, mean arterial pressure; HR, heart rate; EtCO_2_, end-tidal CO_2_. Different superscript letters denote significant differences between groups, *p* < 0.05.

### 3.4 Blood gas among the three groups

The data of blood gas among the three groups represented that the pH in Group P was higher than that in Group G *p* < 0.05 at A1. The value of HCO_3_^−^ in Group P and Group V were higher than those in Group G *p* < 0.05 at A2 (Table 4).

**Table 4:** Comparison of blood gas values in the three groups at different time points.

Variables	Group V	Group P	Group G	*P-*value	Statistic (F/χ^2^)
A1					
pH	7.27 ± 0.05^ab^	7.29 ± 0.05^b^	7.26 ± 0.05^a^	0.046	3.184
O_2_ (mmHg)	165.38 ± 65.65^a^	199.56 ± 57.17^a^	180.32 ± 87.63^a^	0.146	1.964
CO_2_ (mmHg)	43.32 ± 6.5^a^	43.28 ± 5.16^a^	44.84 ± 7.32^a^	0.506	0.686
HCO_3_^−^ (mmol/L)	19.31 ± 2.23^a^	20.03 ± 0.91^a^	19.4 ± 2.11^a^	0.164	1.129
A2					
pH	7.26 ± 0.12^a^	7.27 ± 0.09^a^	7.29 ± 0.08^a^	0.555	0.593
O_2_ (mmHg)	146.79 ± 58.68^a^	151.65 ± 69.51^a^	143.29 ± 70.62^a^	0.867	0.143
CO_2_ (mmHg)	46.36 ± 23.23^a^	45.27 ± 13.91^a^	39.25 ± 12.61^a^	0.179	1.751
HCO_3_^−^ (mmol/L)	18.99 ± 2.59^b^	19.31 ± 3.04^b^	17.48 ± 3.73^a^	0.038	3.389
A3					
pH	7.27 ± 0.13^a^	7.29 ± 0.09a	7.29 ± 0.82^a^	0.643	0.443
O_2_ (mmHg)	157 ± 69.05^a^	170.03 ± 74.67^a^	159.62 ± 81.32^a^	0.338	1.095
CO_2_ (mmHg)	44.01 ± 26.68^a^	39.24 ± 10.46^a^	38.48 ± 12.26^a^	0.381	0.973
HCO_3_^−^ (mmol/L)	17.88 ± 3.22^a^	17.84 ± 2.89^a^	17.42 ± 2.52^a^	0.758	0.278

pH, potential of hydrogen; O_2_, oxygen; CO_2_, carbon dioxide; HCO_3_-, bicarbonate. Different superscript letters denote significant differences between groups, *p* < 0.05. Serious intraoperative cardiopulmonary adverse events (*N* = 105) included elevated airway pressure >20 cmH_2_O (*n* = 17), hypoxemia with SpO_2_ < 90% (*n* = 4), hypotension (*n* = 18), and intravenous epinephrine administration (*n* = 23).

Among them, eight patients in the Group V showed increased peak airway pressure (PIP > 20 cmH_2_O), two at time point T2, five at time point T3, and one at time point T5. An increase in airway pressure occurred in two cases in the Group P, both at T3. Airway pressure increased in seven patients in the Group G: one at T2, two at T3, three at T4, and one at T5. The immediate airway pressure (T0) after intubation was less than 20 cmH_2_O in all groups.

Hypoxemia occurred in two patients in the Group P, one patient each in the Group G, and Group V.

Blood pressure decreased in 8 patients in the Group P, 6 in the Group G, and 4 in the Group V.

## 4 Discussion

The treatment landscape for retinoblastoma has evolved significantly over the years, with a shift toward localized therapies such as selective ophthalmic arterial chemotherapy (SOAC) due to its efficacy and reduced systemic toxicity compared to conventional systemic chemotherapy (17). Our study delved into the perioperative management of children with retinoblastoma undergoing SOAC, focusing specifically on the choice of ventilation mode during the procedure. In this study, we compared the intraoperative effects of three different respiratory ventilation modes including VCV, PCV and PCV-VG during SOAC under general anesthesia, and the results indicated that the PCV and PCV-VG modes provided lower peak airway pressure during SOAC, and the PCV mode was more conducive to maintaining the stability of EtCO_2_ and normal respiratory-related metabolic indicators in blood gas results. These findings would help select the optimal method of general anesthesia for children with retinoblastoma.

One of the key challenges in administering SOAC to pediatric patients is the perioperative management of anesthesia, particularly due to the physiological and anatomical differences between children and adults. SOAC usually requires general anesthesia due to the generally young age of retinoblastoma children. However, severe respiratory and circulatory complications have been frequently reported in children undergoing this type of surgery since 2011 (7, 10). At present, the specific cause of these potential complications remains unclear, but an acute decline in pulmonary compliance may be the initial factor leading to the development of hypoxemia and hypotension in children (18). Due to tracheal stenosis and small lung volume in children, an acute decrease in lung compliance is more likely to cause a sharp increase in airway pressure and mechanical lung injury.

Common ventilation modes for general anesthesia in infants and children are VCV, PCV, and PCV-VG. The PCV-VG mode integrates the characteristics of the VCV and PCV modes (19). It provides a stable tidal volume while maintaining the airway pressure above a preset value; it also ensures ventilation and oxygen delivery in pediatric patients (20). In this study, the average airway pressure in Group V was significantly higher than that in the Group P and Group G. The patients with intraoperative airway pressure >20 cmH_2_O were mainly in the Group V and Group G, among which eight patients in the Group V had increased airway pressure >20 cmH_2_O, seven patients in the Group G had increased airway pressure, and only two patients in the Group P had increased airway pressure. Interestingly, almost all cases of airway pressure increase occurred within 5 min after microcatheter placement and contrast agent injection. A report published in 2019 described a pediatric patient undergoing SOAC surgery who experienced a rapid decrease in tidal volume of 75% as the catheter passed through the carotid cavernous sinus and reached the ophthalmic artery. This confirmed the findings of previous studies that super-SOAC, when a microcatheter is placed between the internal carotid artery and the ophthalmic artery, is prone to induce reflex, since this area is innervated by the trigeminal nerve, it was inferred that a trigeminal reflex was triggered, resulting in decreased lung compliance and increased airway pressure (21). However, PCV applies airway pressure values to limit the VCV, and the pressure can change synchronously according to lung compliance. Therefore, according to the study results, the airway pressure change range of children in the Group P was too small to avoid possible lung injury caused by an acute airway pressure increase resulting from a sudden decrease in lung compliance. However, although a temporary increase in airway pressure is avoided, it is unclear whether the PCV mode meets the normal ventilation and oxygenation requirements of children after limiting the airway pressure. Furthermore, it does not seem to affect the acid–base balance of the children’s vital signs and blood gas indices in the case of a sharp decline in lung compliance.

This study found no significant differences in heart rate or blood pressure among the three groups at different time points. Previous studies have found that approximately 34% of children experienced a drop in carbon dioxide of >30% during treatment (9), and this may be due to a sudden increase in pulmonary vascular resistance in children; although there is still no strong evidence to prove this. However, our study also found that the mean value of EtCO_2_ in the Group V and Group G decreased gradually after microcatheter placement in the internal carotid artery, but the EtCO_2_ in the Group P remained relatively stable. If the so-called reflex in SOAC is a decrease in lung compliance due to bronchospasm caused by hypersensitivity, it cannot explain the decrease in carbon dioxide. However, if an increase in pulmonary vascular resistance is considered, maintaining the original tidal volume for a short period may further aggravate the imbalance in the ventilated blood flow ratio in children. Whenever a decrease in carbon dioxide occurs, and a pressure ventilation mode is used, while limiting peak airway pressure, this appears to be more conducive to stabilizing EtCO_2_ values in children with reduced lung ventilation and pulmonary blood flow. Although statistically significant improvements in airway pressures and gas exchange parameters were observed in the Group P and Group G, these values largely remained within clinically safe physiological ranges, their clinical superiority in terms of reducing major perioperative morbidity remains suggestive. Future large-scale studies involving patients with pre-existing pulmonary pathology are required to determine whether these physiological benefits translate into improved hard clinical outcomes.

Further from the blood gas analysis value, the mean pH value of the Group V at T1 was lower than that of the other two groups. PCV mode can better maintain the respiratory index and acid–base balance in children. Five minutes after the contrast agent injection, the bicarbonate value in the Group P was closer to the normal value in the Group G. This is also consistent with the changes in the EtCO_2_ values of the children in this study. It seems that PCV mode can better adapt to the so-called “sudden decline in lung compliance” that may occur in children during the operation and can better adapt to the imbalance of lung ventilation blood flow ratio, so as to maintain stable vital signs and internal environment of children (22).

This study has several limitations. First, the retrospective design and relatively small sample size inherently carry a risk of selection bias. Second, owing to this retrospective nature and the short, minimally invasive profile of the SOAC procedure, our ability to comprehensively evaluate postoperative outcomes was restricted. Relying on standard anesthesia records precluded the use of granular, validated scoring systems for emergence agitation and postoperative nausea and vomiting (PONV). Additionally, because postoperative chest radiographs and inflammatory markers were not routinely assessed, we were unable to evaluate subclinical inflammatory responses or long-term postoperative pulmonary complications. Third, to minimize statistical confounding, we excluded patients who developed severe intraoperative complications requiring manual ventilation—events often accompanied by acute airway pressure spikes, hemodynamic instability, or oxygen desaturation that preclude accurate machine monitoring. Consequently, our findings may not be fully generalizable to critically ill pediatric populations, a demographic for whom the choice of mechanical ventilation mode might have a particularly profound clinical impact. Despite these limitations, this study provides valuable clinical insights into intraoperative ventilation management. Future prospective trials incorporating validated scoring tools and comprehensive clinical assessments are warranted to fully elucidate the holistic postoperative benefits of these ventilation modes (particularly PCV-VG), explore the mechanisms of cardiopulmonary complications, and establish optimal intraoperative crisis-response strategies.

Altogether, this study compared the intraoperative effects of three different respiratory ventilation modes including VCV, PCV and PCV-VG during SOAC under general anesthesia. Our results demonstrated that while all three modes successfully maintained the end-tidal partial pressure of carbon dioxide and blood gas indices within normal physiological ranges, the mean airway pressure in the PCV mode was lower than that in the VCV and PCV-VG modes. This suggests that PCV can effectively provide lung protection by lowering airway pressures while reliably maintaining internal environmental stability and acid–base balance.

## 5 Conclusion

The mean airway pressure in the PCV mode was lower than that in the VCV and PCV-VG modes, and the end-expiratory partial pressures of carbon dioxide and blood gas analysis indices were closer to normal values, showing more effective lung protection and maintenance of internal environmental stability and acid–base balance.

## Data Availability

The raw data supporting the conclusions of this article will be made available by the authors, without undue reservation.
